# Analytical Performance Validation of Next-Generation Sequencing Based Clinical Microbiology Assays Using a K-mer Analysis Workflow

**DOI:** 10.3389/fmicb.2020.01883

**Published:** 2020-08-05

**Authors:** Sarah Lepuschitz, Thomas Weinmaier, Katharina Mrazek, Stephan Beisken, Johannes Weinberger, Andreas E. Posch

**Affiliations:** Ares Genetics GmbH, Vienna, Austria

**Keywords:** antimicrobial resistance, whole genome sequencing, human pathogens, workflow validation, k-mer analysis

## Abstract

Next-generation sequencing (NGS) enables clinical microbiology assays such as molecular typing of bacterial isolates which is now routinely applied for infection control and epidemiology. Additionally, feasibility for NGS-based identification of antimicrobial resistance (AMR) markers as well as genetic prediction of antibiotic susceptibility testing results has been demonstrated. Various bioinformatics approaches enabling NGS-based clinical microbiology assays exist, but standardized, computationally efficient and scalable sample-to-results workflows including validated quality control parameters are still lacking. Bioinformatics analysis workflows based on k-mers have been shown to allow for fast and efficient analysis of large genomics data sets as obtained from microbial sequencing applications. We here demonstrate applicability of k-mer based clinical microbiology assays for whole-genome sequencing (WGS) including variant calling, taxonomic identification, bacterial typing as well as AMR marker detection. The wet-lab and dry-lab workflows were developed and validated in line with Clinical Laboratory Improvement Act (CLIA) guidelines for laboratory-developed tests (LDTs) on multi-drug resistant ESKAPE pathogens. The developed k-mer based workflow demonstrated ≥99.39% repeatability, ≥99.09% reproducibility and ≥99.76% accuracy for variant calling and applied assays as determined by intra-day and inter-day triplicate measurements. The limit of detection (LOD) across assays was found to be at 20× sequencing depth and 15× for AMR marker detection. Thorough benchmarking of the k-mer based workflow revealed analytical performance criteria are comparable to state-of-the-art alignment based workflows across clinical microbiology assays. Diagnostic sensitivity and specificity for multilocus sequence typing (MLST) and phylogenetic analysis were 100% for both approaches. For AMR marker detection, sensitivity and specificity were 95.29 and 99.78% for the k-mer based workflow as compared to 95.17 and 99.77% for the alignment-based approach. Summarizing, results illustrate that k-mer based analysis workflows enable a broad range of clinical microbiology assays, potentially not only for WGS-based typing and AMR gene detection but also genetic prediction of antibiotic susceptibility testing results.

## Introduction

Infections caused by antibiotic resistant bacteria are one of the most serious public health challenges worldwide. Due to overuse and misuse of antibiotics, previously manageable bacterial infections are becoming hard-to-treat (WHO, 2014). In order to effectively address this challenge, fast and comprehensive diagnostic information prior to treatment is of utmost importance (Leekha et al., 2011).

Whole genome sequencing (WGS) of bacterial isolates can give access to detailed information about taxonomic classification, genomic variations, chains of transmission and the presence of antimicrobial resistance (AMR) or virulence markers. Already today, WGS is used to inform infection control management, enhance molecular epidemiology efforts and identify unknown organisms (Gargis et al., 2012). Reproducibility and accuracy of WGS-based microbial typing has already been demonstrated by different laboratories (Kozyreva et al., 2017; Yachison et al., 2017; Portmann et al., 2018; Bogaerts et al., 2019) and the Clinical and Laboratory Standards Institute (CLSI) recently published guidelines (Clinical and Laboratory Standards Institute [CLSI], 2014) including recommendations for the use of NGS for infectious disease testing applications.

In addition to accurate microbial typing, WGS can also enable genetic detection of AMR markers as well as genetic prediction of antibiotic susceptibility testing (AST) results using machine learning (Su et al., 2019). Recently, several studies have demonstrated feasibility and potential for next-generation sequencing based prediction of AST results (Gordon et al., 2014; Bradley et al., 2015; Clausen et al., 2016; Pesesky et al., 2016; Tamma et al., 2019; Volz et al., 2019; Ferreira et al., 2020). Different bioinformatics workflows have been developed to identify AMR markers based on curated AMR databases including CARD RGI (Jia et al., 2017) and AMRfinderPlus (Feldgarden et al., 2019). In this study, we describe AMR marker detection based on ARESdb, a curated AMR reference database linking AMR markers to diagnostic performance indicators for association with phenotypic resistance based on matched WGS-AST data from more than 50,000 isolates. AMR markers, as accessible via the QIAGEN CLC Microbial Genomics ARESdb Module with associated performance indicators, were used in this study.

**FIGURE 1:**
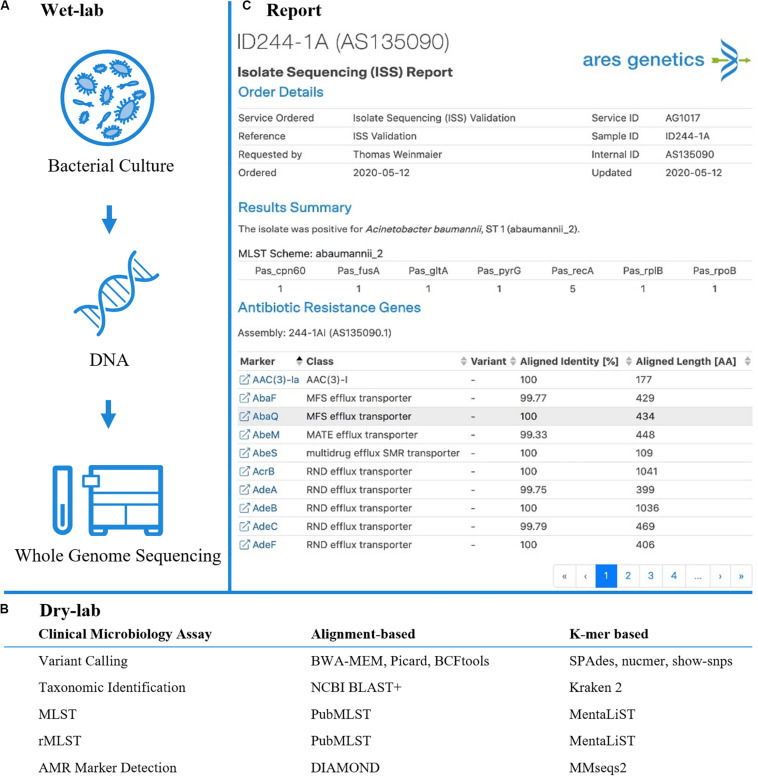
Established and validated workflow for WGS from bacterial isolates. **(A)** A state-of-the-art wet-lab workflow for processing of bacterial isolates was implemented. **(B)** Dry-lab analysis of WGS data was evaluated using alignment-based and k-mer based bioinformatics tools for clinical microbiological assays (including variant calling, taxonomic identification, MLST, rMLST, AMR marker detection). For AMR marker detection, AMR markers with associated performance indicators were used as accessible via the QIAGEN CLC Microbial Genomics ARESdb Module (https://resources.qiagenbioinformatics.com/manuals/clcmgm/current/index.php?manual = ARES_Database.html). **(C)** The analysis report as provided via *ares-genetics.cloud*, including results for taxonomic identification, subtyping and AMR marker detection (illustrated for validation sample ID244-1A).

The present study focusses on the validation of k-mer based workflows as the application of k-mer based approaches for microbial bioinformatics is widespread and tools like Kraken (Salzberg and Wood, 2014) have been shown to facilitate precise taxonomic classification even in presence of contaminating sequences or low sequencing depth (Wood et al., 2019). Additionally, k-mer based workflows have been found to detect AMR markers from NGS at high sensitivity and specificity (Clausen et al., 2016) and have been shown to enable genetic antibiotic susceptibility prediction using machine learning (Aun et al., 2018; Su et al., 2019). To further advance the translation of k-mer based workflows from research to clinical microbiology applications, we here describe the development and validation of a k-mer based WGS workflow by comparison to a state-of-the-art alignment based workflow for clinical microbiology assays including variant calling, taxonomic identification, bacterial typing and AMR marker detection. The wet-lab and dry-lab workflows were developed and validated in line with Clinical Laboratory Improvement Act (CLIA) guidelines for laboratory-developed tests (LDTs) on multi-drug resistant ESKAPE pathogens. The study systematically validates k-mer based clinical microbiology assays in comparison to alignment-based workflows for the first time (Figure 1).

## Materials and Methods

### Definition of Intended Use

The developed WGS isolate sequencing workflow is intended for bacterial isolates (Biosafety Risk group 1 and 2) retrieved from patients with an infectious disease. Paired-end WGS from a pure bacterial culture performed on Illumina platforms (Illumina, San Diego, CA, United States) is a non-targeted sequencing approach of randomly fragmented genomic DNA and does not use *a priori* knowledge of sequence targets. Bioinformatics pipelines include all required steps from raw sequencing data to data output for clinical infectious disease applications including taxonomic identification/confirmation, phylogenetic relationships and detection of antimicrobial resistance markers.

### Validation Plan

Validation procedures were based on previously published studies (Rehm et al., 2013; Gargis et al., 2016; Kozyreva et al., 2017). Definitions and metrics for analysis were applied and performed according to Kozyreva et al. (2017), providing a modular template for the validation of WGS-processes in microbiology laboratories according to CLIA guidelines. The analytical validation comprised three phases: workflow development, workflow validation and quality management (Supplementary Figure 1).

Workflow development lasted 6 months with extensive testing, iteration and optimization of sample processing (microbial cultivation of diverse bacterial species, DNA extraction from Gram positive/negative bacteria, library preparation and pooling for WGS, testing of different Illumina sequencing chemistries/platforms and bioinformatics analysis tools) including the setup of standard operating procedures (SOPs).

Workflow validation was executed end-to-end from sample preparation via whole genome sequencing to bioinformatics data analysis with representative human ESKAPE pathogens (validation set). All steps were validated for alignment and k-mer based workflows by assessing: (i) accuracy of the platform (quality parameters respectively variant calling), clinical microbiology assays (Figure 1) for taxonomic identification (16S rRNA identification, Kraken2), bacterial typing [MLST, ribosomal (r)MLST], AMR marker detection and genotyping (phylogenetic analysis); (ii) within-run precision (repeatability) and between-run precision (reproducibility) for variant calling and applied assays; (iii) analytical sensitivity (LOD for applied assays) and specificity (contamination analysis); (iv) diagnostic sensitivity and specificity (MLST and genotyping).

Finally, based on assay validation results, quality control measures were developed to identify sample-preparation failures as well as measures to identify failed sequencing runs.

### WGS Wet Lab Workflow

#### Bacterial Isolates and Reference Materials

To validate the workflow, a set of seven bacterial species known to exhibit multidrug resistance were processed from cultivation to sequence analysis. Bacterial isolates with high-quality public reference sequences were selected and included all ESKAPE pathogens (*Enterococcus faecium*, *Staphylococcus aureus*, *Klebsiella pneumoniae*, *Acinetobacter baumannii*, *Pseudomonas aeruginosa*, *Enterobacter cloacae*) (Rice, 2008) and *Escherichia coli*. Finished, high quality reference genomes were either retrieved from NCBI RefSeq or the ATCC Genome Portal [available at genomes.atcc.org (Accessed: 12 February 2020)] (Table 1).

**TABLE 1:** Set of bacterial strains used for internal WGS workflow validation.

Internal sample ID	Reference species	Reference ID	Database (accession)
ID244	*Acinetobacter baumannii*	ATCC BAA-1605	ATCC Genome Portal (n.a.)
ID245	*Pseudomonas aeruginosa*	ATCC 27853	ATCC Genome Portal (n.a.)
ID246	*Klebsiella pneumoniae*	ATCC 700603	ATCC Genome Portal (n.a.)
ID247	*Staphylococcus aureus*	ATCC BAA-2312	ATCC Genome Portal (n.a.)
ID248	*Escherichia coli*	ATCC 35218	ATCC Genome Portal (n.a.)
ID249	*Enterococcus faecium*	ATCC 700221	RefSeq (GCF_001594345.1)
ID250	*Enterobacter cloacae*	NCTC 13464	RefSeq (GCF_900447465.1)

#### Microbial Cultivation and DNA Isolation

Bacterial isolates were cultivated overnight according to the propagation procedure of the supplier (Microbiologics, MN, United States). Automated DNA extraction from Gram-positive and Gram-negative bacteria was performed on a QIAsymphony instrument (QIAGEN, Hilden, Germany) using the QIAsymphony DSP DNA Kit (QIAGEN). Modifications of the manufacturers standard protocol included: for extraction from *S. aureus* addition of lysostaphin solution (5 U per sample; Sigma-Aldrich, St. Louis, MO, United States); lysis of Gram-positive bacteria on device (37°C/shaking at 900 rpm for 1 h); a third wash step to enhance purification for extraction of Gram-positive and Gram-negative bacteria. Each independent DNA extraction contained a no template control (NTC) containing molecular grade water only.

#### Library Preparation and WGS Sequencing

The purity of isolated DNA was determined via A260/A280 ratios on a QIAxpert (QIAGEN) UV/VIS spectrophotometer and quantified on a Quantus Fluorometer (Promega, MI, United States) using QuantiFluor dsDNA Dye (Promega).

To assess repeatability/reproducibility each validation sample (*n* = 7) was sequenced in triplicate within one sequencing run (NextSeq, run A) as well as on two additional single runs (MiSeq, run B and C). For within-run precision, three sample replicates (1AI-1AIII) starting from the same DNA extract were included in independent library preparations (operator I-III) and were sequenced under identical conditions. For between-run precision, three sample replicates (1AI, 2B, 3C) were generated from fresh overnight cultures and processed by one operator. Consequently five replicates of each validation sample were sequenced (Supplementary Figure 3).

NGS libraries were prepared according to the manufacturer’s instructions using QIAseq FX DNA Library Kit (QIAGEN). Library concentration was determined in the same manner as the input DNA concentration and library fragment analysis was carried out on a QIAxcel System (QIAGEN). Paired-end sequencing was performed on Illumina MiSeq or NextSeq instruments (Illumina, San Diego, CA, United States) using MiSeq 300-cycle Reagent Micro Kit v2 respectively NextSeq 300-cycle Reagent Mid Out-put Kit (Illumina). Each independent library preparation contained a NTC starting from the DNA extraction.

### WGS Dry Lab Workflow

For each of the clinical microbiology assays an alignment and a k-mer based analysis tool were compared. To more generally confirm robustness of dry lab workflows, results from primary tools were compared against supplementary state-of-the-art tools.

#### Raw Data Processing and Assembly

Sequencing reads (FASTQ) were quality trimmed and filtered using Trimmomatic v0.39 (Bolger et al., 2014) with parameters “ILLUMINACLIP:adapters.fa:2:30:10 LEADING:10 TRAILING:10 SLIDINGWINDOW:4:15 MINLEN:36.” The file adapters.fa has been compiled from standard Illumina adapters. Filtered reads were *de novo* assembled using SPAdes v3.13.1 (Bankevich et al., 2012) with parameter “–careful” and then annotated using Prokka v1.14.1 (Seemann, 2014). Completeness of the assembled genomes was assessed using BUSCO v3 and set of bacterial orthologues (Waterhouse et al., 2018).

#### Variant Calling

For alignment based variant calling, filtered reads from each species were aligned to the corresponding reference genome using bwa-mem v0.7.17 (Li, 2013) and the resulting alignments were sorted and duplicates marked using the functions “SortSam” and “MarkDuplicates” from Picard v2.21.2^1^. Variants were then called using bcftool v 1.9 + htslib-1.9^2^ and filtered using vcftools with parameters “–minQ 200 –remove-indels”(Danecek et al., 2011). To confirm results, filtered reads from each species were also aligned to the corresponding reference genome also using minimap2 v2.17-r974-dirty and parameters “-ax sr.”

For the k-mer based approach, SPAdes assembled genomes (parameters as described above) were compared to the reference genome using nucmer v3.1 (Kurtz et al., 2004) and variants were called using show-snps (Kurtz et al., 2004). Variant counts for both approaches were determined using custom scripts.

#### Taxonomic Identification

For alignment based 16S rRNA identification, assembled contigs were compared against a custom 16S rRNA database derived from RefSeq (access date 2019-09-19) (O’Leary et al., 2016) using ncbi-blastn v2.9.0+ (Camacho et al., 2009) and the top hit was used for taxonomic assignment.

K-mer based Kraken 2 v2.0.8-beta (Wood et al., 2019) with default settings was executed on the filtered reads and the species with the highest proportion of assigned reads was picked as taxonomic assignment. Results of Kraken2 were confirmed using KrakenUniq v0.5.8 (Breitwieser et al., 2018) with settings “–fastq-input –gzip-compressed –preload –paired –check-names.” The KrakenUniq reference database was generated on June 30th from complete bacterial and archaeal genomes in RefSeq according to instructions in the KrakenUniq GitHub repository.

#### Bacterial Typing

For alignment based multi locus sequence typing (MLST), sequence types (ST) were extracted from WGS-data (Jolley and Maiden, 2010) from PubMLST databases^3^ for *E. faecium*, *S. aureus*, *A. baumannii*, *P. aeruginosa*, Enterobacter spp. and from Institute Pasteur MLST databases^4^ for *K. pneumoniae* and *E. coli* using the tool mlst^5^.

Determination of alignment based universal ribosomal MLST (rMLST) was performed by sequence queries of the assembled replicates against the rMLST database (Jolley et al., 2012)^6^.

K-mer based MLST analysis was performed using MentaLiST (Feijao et al., 2018) and MLST profiles from PubMLST. For k-mer based rMLST, MentaLiST was used to generate a custom database from marker sequences and profiles from the rMLST database and assembled replicates were searched against this custom database. The MentaLiST codebase was adjusted to be able to deal with large allele counts by changing variable datatypes “Int16” to “Int32.”

Additional verification of MLST results was performed directly from raw reads using the tool MLST 2.0 [Software version: 2.0.4 (2019-05-08) Database version: 2.0.0 (2020-06-22)] (Larsen et al., 2012) available from the Center of Genomic Epidemiology (CGE)^7^.

#### Antibiotic Resistance Genes Detection

Alignment based detection of AMR resistance markers was performed by 6-frame translation of the assembled genome and comparing all translated open reading frames against the QIAGEN CLC Microbial Genomics ARESdb Module marker reference database using Diamond (Buchfink et al., 2014) with a minimal query coverage of 60% and a minimal identity of 90%. For the k-mer based approach, proteins annotated by Prokka (Seemann, 2014) were compared to the AMR marker reference database using mmseqs2 (Steinegger and Söding, 2017) with minimal query coverage of 60% and a minimal identity of 90%. Evaluation was carried out against a ground truth set of AMR marker hits that was identified by comparing the annotated proteins from the seven reference genomes against the AMR marker reference database using ncbi-blast v2.9.0 + with parameter “qcov_hsp_perc 60” and subsequent filtering of the blast hits to a minimal identity of 90%.

#### Phylogenetic Analysis

To determine the genotyping accuracy (alignment- and k-mer based) via genetic relatedness, three (ID244, ID247, ID248) out of seven validation samples were randomly selected to calculate each validation tree with a concordant reference tree. According to Kozyreva et al. (2017), validation trees comprised five sequences including the validation sequence plus four reference sequences (Supplementary Table 6) of the respective species retrieved from the National Center for Biotechnology Information (NCBI) Genome database. For reference trees the validation sequence was replaced by the original reference sequence.

The whole genome alignment of the assembled reference genomes was generated using MAUVE v2.4.0 (Darling et al., 2010) and a phylogenetic tree was calculated using RAxML v8.2 (Stamatakis, 2014) with parameters “-m ASC_GTRCAT -p 12345 -# 100 -b 12345.”

For k-mer based analysis, Sourmash (Pierce et al., 2019) was used with parameters “-k 31 –scaled 1000” to compute distances and calculate a dendrogram. The topological similarity and agreement of clustering patterns was determined by visual comparison of validation and reference trees.

Additional verification of phylogenetic analysis based on raw read alignment was performed using Snippy version 3.2-dev^8^, which uses BWA-MEM v0.7.17-r1188 for short read mapping and Freebayes v1.3 for SNP calling. A phylogenetic tree was calculated using RAxML v8.2 as described above.

#### Definitions for Accuracy and Repeatability/Reproducibility

Definitions and calculations for accuracy and repeatability/reproducibility as described by Kozyreva et al. (2017) were used and adapted for platform accuracy for base calling (precision for variant positions) (Supplementary Table 1).

#### Analytical Sensitivity, Limit of Detection (LOD), and Specificity

The analytical performance of applied alignment and k-mer based assays (taxonomic identification, bacterial typing, AMR marker detection) was assessed by downsampling of all validation samples. Required read counts for sequencing depths of 90×, 80×, 70×, 60×, 50×, 40×, 30×, 20×, 15×, 10×, and 5× and 150 bp paired-end sequencing were determined based on the size of the reference genome for each species. The seqtk^9^ function “sample” was used with parameter “-s100” to subsample reads starting from each replicate of each species and the resulting subsampled read set was used as input for the following subsample. SNPs were called as described above and the LOD for variant calling was determined by manual inspection. LOD for marker detection was defined as sensitivity and specificity ≥85%.

Further, analytical specificity was assessed by mimicking *in silico* contamination of one validation sample by the addition of reads from different samples (identical and different species). One randomly selected validation sample (ID245, *P. aeruginosa*) was set as “original” sample. “Mixture” samples contained equal parts of reads from the original and from other samples [ID244, ID246, ID247, ID248, ID249, ID250 and *P. aeruginosa* (SRR8377272)] and were downsampled to a sequencing depth of 90× using seqtk. Contamination analysis was performed using Kraken 2 and KrakenUniq as described above. The “original” sample was compared to “mixture” samples based on assembly size, L50 value, percentage of genome duplication and the presence of 1st/2nd prevalent genera to estimate analytical specificity.

#### Diagnostic Sensitivity and Specificity

Diagnostic sensitivity and specificity were assessed for alignment and k-mer based MLST and genotyping. For interpretation, results are defined as likelihoods and had to be classified either as true positives (TP), false positives (FP), true negatives (TN), or false negatives (FN) in comparison to the reference sequence, which was done in concordance to recommendations by Kozyreva et al. (2017). All validation assemblies were queried against MLST databases from matching- (TP/FN) and five (ID244, ID245, ID246, ID248, ID249) randomly selected from non-matching (TN/FP) species (Supplementary Tables 16, 17). For genotyping, the agreement (TP/TN) and disagreement (FP/FN) of clustering patterns between alignment and k-mer based validation and reference trees was determined (Supplementary Table 18). Sensitivity for AMR marker detection was determined based on the ratio of numbers of markers detected in the validation data set divided by the number of correctly identified markers in the reference genome. Specificity for AMR marker detection was determined based on the ratio of true negative AMR markers as identified by WGS divided by the number of AMR markers found to be absent in the finished reference genome.

### Quality Management

To ensure quality and consistency of routine sample processing, quality control (QC) was performed throughout wet-lab and dry-bench processes. QC steps were preliminarily based on previous sequencing experience and recommendations from literature after DNA isolation (concentration and purity), library preparation (concentration and size distribution), sequencing (Q30 score, cluster density, cluster passing filter, PhiX error rate), for raw sequencing data (minimum read pair count per sample, minimum read length after trimming) and data analysis (# unique rRNAs, # unique tRNAs, estimated genome duplication rate and genome completeness, L50 and N50 for *de novo* assembly, minimum coverage 20×, if available concordance of taxonomic identification with submitter ID). NTCs (*n* = 5) were processed as negative controls according to the sample preparation plan (Supplementary Figure 3) to control contamination during DNA isolation, library preparation and sequencing. As error rate control a ready-to-use PhiX v3 (Illumina) library was used according to the manufacturer’s instructions (Illumina).

## Results

Key analytical performance validation results across clinical microbiology assays are summarized in Table 2.

**TABLE 2:** Summarized assay accuracy results for validation samples and retrieved reference sequences.

Sample ID	Reference species	Alignment based assays				k-mer based assays			
			
		Taxonomic identification	Bacterial typing		Marker detection	Taxonomic identification	Bacterial typing		Marker detection
							
		16S rRNA identification	MLST	rMLST/Species	AMR gene detection (val/ref)	Kraken 2	MLST	rMLST/Species	AMR gene detection (total val/ref)
ID244	*A. baumannii*	*A. baumannii*	ST1	rST8954/ *A. baumannii*	178/191	*A. baumannii*	ST1	rST8954/ *A. baumannii*	179/191
ID245	*P. aeruginosa*	*P. aeruginosa*	ST155	rST20748/ *P. aeruginosa*	86/86	*P. aeruginosa*	ST155	rST20748/ *P. aeruginosa*	86/86
ID246	*K. pneumoniae*	*K. pneumoniae*	ST489	rST19205/ ***K. quasipneumoniae***	345/357	***K. quasipneumoniae***	ST489	rST19205/ ***K. quasipneumoniae***	346/357
ID247	*S. aureus*	*S. aureus*	ST130	rSTnew/ *S. aureus*	115/116	*S. aureus*	ST130	rSTnew/*S. aureus*	115/116
ID248	*E. coli*	*E. coli*	ST33	rST2194/ *E. coli*	332/333	*E. coli*	ST33	rST2194/ *E. coli*	331/333
ID249	*E. faecium*	*E. faecium*	ST17	rST18445/E. faecium	17/19	*E. faecium*	ST17	rST18445/E. faecium	17/19
ID250	*E. cloacae*	*E. cloacae*	ST278	rST71024/ ***E. hormaechei***	265/301	***E. hormaechei***	ST278	rST71024/ ***E. hormaechei***	266/301
Average Accuracy (%)		100%	100%	100%	99.76%	100%	100%	100%	99.76%

*For taxonomic identification respectively bacterial typing, alignment as well as k-mer based assays revealed 100% accuracy determined by the agreement between validation and reference sequences. AMR gene detection revealed an average accuracy of 99.1 and 99.9% for alignment based respectively kmer based assays. rSTnew = a novel rMLST for ID247 was identified due to a new combination of alleles (val/ref) = number of correctly identified AMR markers in the validation sequence/total number of AMR markers detected in the reference sequence. Bold species names indicate assay results that differed from the reference genome species classification.*

### Determination of Platform and Assay Accuracy

Accuracy of the platform was determined by the agreement between variant calling of the validation and the reference sequence and revealed an agreement of 99.99–100% for alignment based and 99.98–100% for k-mer based variant calling respectively. Similarly, Minimap2 yielded 100% accuracy for variant calling (Supplementary Table 1).

Assay accuracy for alignment and k-mer based taxonomic identification (16S rRNA identification, Kraken 2), bacterial typing (MLST, rMLST) and phylogenetic analysis revealed 100%. Verification of taxonomic identification, bacterial typing and phylogenetic analysis by KrakenUniq, CGE MLST and Snippy revealed 100% accuracy as well (Supplementary Tables 2–4, 6 and Supplementary Figure 4). AMR marker detection revealed an average accuracy of 99.76% for alignment and k-mer based workflows (Supplementary Table 5). For two out of seven samples (ID246, ID250) the taxonomic identification via 16S rRNA identification and MLST was confirmed but Kraken2, KrakenUniq and rMLST reassigned previously defined species from database records. ID246 (*Klebsiella pneumoniae*) was therefore assigned to *Klebsiella quasipneumoniae* and ID250 (*Enterobacter cloacae*) to *Enterobacter hormaechei*.

### Repeatability and Reproducibility

Repeatability and reproducibility were assessed by running multiple samples under identical conditions (within-run) and under changed conditions (between-run). Precision of quality metrics for all five replicates per sample are listed in Supplementary Table 7. Variant calling was evaluated relative to the reference genome size and revealed an alignment based repeatability of 99.98% and reproducibility of 99.98% and k-mer based repeatability of 98.35% and reproducibility of 99.41%. Verification of results using minimap2 revealed 99.99% repeatability and 99.99% reproducibility (Supplementary Table 8).

Assays for alignment and k-mer based taxonomic identification (16S rRNA identification, Kraken 2) respectively bacterial typing (MLST, rMLST) showed 100% repeatability and 100% reproducibility. Similarly, KrakenUniq and CGE MLST yielded 100% repeatability and 100% reproducibility (Supplementary Tables 9–11). The agreement of alignment based AMR marker detection resulted in 99.49% repeatability and 99.20% reproducibility, k-mer based AMR marker detection resulted in 99.39% repeatability and 99.09% reproducibility (Supplementary Table 12). Bioinformatics pipeline iterations revealed consistent results and confirmed reproducibility of raw data processing and analysis.

### Analytical Sensitivity and Specificity

Analytical sensitivity showed a minimum sequencing depth for alignment and k-mer based taxonomic identification of 10× and 5×, for bacterial typing 15× and 20×, for AMR marker detection 15× and 15×. Re-examination of minimum sequencing depths for taxonomic identification (KrakenUniq) and bacterial typing (CGE MLST) revealed 5× and 40×, respectively (Supplementary Table 13).

Specificity was determined by mimicking *in silico* contamination of validation sample ID245 (P. aeruginosa) with raw data (identical and different species). Comparison of the “original” sample with “mixture” samples including discordant species revealed an increase of assembly size from 6.91 Mb to 9.80–12.26 Mb, L50 from 6 to 14–22 and genome duplication from 0% to 79.70–90.50%. The most abundant genus in the “original” samples was Pseudomonas spp. (97.04%), which decreased down to 46.98–47.84% in “mixture” samples with discordant species. “Mixture” samples with discordant species revealed the second most abundant genus to be in concordance with prior taxonomic identification of contaminating reads. Contamination of the “original” sample with reads from the identical species (*P. aeruginosa* Pao X1) increased the assembly size from 6.91 to 15.96 Mb, L50 from 6 to 409 and genome duplication from 0 to 43.9%. Pseudomonas spp. remained the most prevalent genus but decreased from 97.04 to 71.62% and revealed Enterobacter spp. to be the second prevalent genus (21.41%). Re-examination of the same dataset using KrakenUniq yielded concordant results (Supplementary Table 14).

### Diagnostic Sensitivity and Specificity

Diagnostic sensitivity was 100% for alignment and k-mer based MLST, defined by the total number of alleles identified correctly (TP, *n* = 50) respectively identified incorrectly (FN, *n* = 0). Diagnostic specificity was 100% for k-mer based MLST of five randomly selected validation sequences, defined by the total number MLST alleles from non-matching species (TN, *n* = 35) respectively the total number of identified alleles in non-matching species (FP, *n* = 0) (Supplementary Tables 15, 16). Diagnostics sensitivity revealed on average 95.17% for alignment based and 99.29% for k-mer based AMR marker detection. Diagnostic specificity revealed on average 99.77% for alignment based and 99.78% for k-mer based AMR marker detection (Supplementary Table 17). Diagnostic sensitivity and specificity for alignment and k-mer based genotyping revealed 100%, by identifying the concordance (TP/TN, *n* = 3) of clustering respectively samples which failed clustering (FP/FN, *n* = 0) between the validation and reference trees (Supplementary Table 18 and Supplementary Figure 4).

### Validation Summary

The observed performance metrics of the validation process are summarized in Table 3. LDT performance parameters per CLIA requirements have to exceed a threshold of ≥90% and was accomplished for all validation steps. Therefore, the successful WGS workflow validation enables a reportable range for alignment and k-mer based taxonomic identification (16S rRNA identification, Kraken2), bacterial typing (MLST, rMLST), AMR marker detection and phylogenetic analysis. The LOD for applied assays was observed at 15× coverage for alignment- respectively at 20× coverage for k-mer based approaches. Verification of applied tools for taxonomic identification (KrakenUniq), genotyping (CGE MLST) and phylogenetic analysis (Snippy) further confirmed robustness of dry-lab workflows.

**TABLE 3:** Summarized results for obtained metrics during the validation. LDT performance parameters per CLIA requirements have to exceed a threshold of ≥90% and was accomplished for all validation steps.

**Performance specification**		**Observed (alignment based)**	**Observed (k-mer based)**
Accuracy	per base	100%	100%
	Taxonomic identification	100%	100%
	Bacterial typing	100%	100%
	AMR marker detection	99.76%	99.76%
	Genotyping	100%	100%
Repeatability (precision within runs)	per base	99.98%	98.35%
	Taxonomic identification	100%	100%
	Bacterial typing	100%	100%
	AMR marker detection	99.49%	99.39%
Reproducibility (precision between runs)	per base	99.98%	99.41%
	Taxonomic identification	100%	100%
	Bacterial typing	100%	100%
	AMR marker detection	99.20%	99.09%
Limit of detection (LOD)	Taxonomic Identification	10×	5×
	MLST	15×	20×
	rMLST	15×	20×
	AMR marker detection	15×	15×
Diagnostic sensitivity	MLST	100%	100%
	AMR marker detection	95.17%	95.29%
	Genotyping	100%	100%
Diagnostic specificity	MLST	100%	100%
	AMR marker detection	99.77%	99.78%
	Genotyping	100%	100%

### Quality Assurance and Quality Control

Quality parameters were defined for tested samples respectively positive/negative controls and implemented via five QC checkpoints for wet-bench (DNA template QC, library QC) and dry-bench processes (run QC, raw data QC, k-mer based analysis QC). The threshold for spiked-in PhiX control was set to an error rate threshold of 0.5–1% and has to be assessed in each run. Reference strain *Escherichia coli* ATCC 35218 was defined to be the monthly positive control, which has to pass all QC checkpoints respectively taxonomic classification must be confirmed by Kraken2, MLST, and rMLST must be assigned to ST33 respectively rST2194 and AMR markers have to be identified at >90% sensitivity and specificity. For phylogenetic analysis, epidemiologically unrelated isolates (outlier) have to be included and should not cluster with tested samples. Negative controls have to be included for each DNA extraction and subsequent library preparation to control contamination. Run QC, raw data QC, analysis QC for negative controls have to be performed on a monthly basis. Precise QC checkpoints for pre-analytical, analytical and post-analytical steps are summarized in Supplementary Table 19. Further all instruments have to be maintained and calibrated according to the manufacturers recommendations. To track sample identity, progress and status throughout the testing process, project specific tracking sheets are organized via an internal, tailored developed framework. Collected data for each sample has to be documented in the respective sample tracking sheet and final results can only be reported if QC results are within the determined range. A scheme describing the established workflow from sample processing to report generation is shown in Supplementary Figure 2.

## Discussion

We here demonstrate applicability of k-mer based WGS workflows for variant calling, taxonomic identification, bacterial typing as well as AMR biomarker detection in comparison with alignment based workflows. The validation process was performed according to CLIA guidelines for laboratory developed tests (LDTs) (Rehm et al., 2013; Gargis et al., 2016; Kozyreva et al., 2017).

Analytical performance of alignment/k-mer based workflows had 99.76/99.76% accuracy, 99.49/99.39% repeatability, 99.20/99.09% reproducibility, 100/100% diagnostic sensitivity and 100/100% diagnostic specificity. Validation results for both, alignment-based and k-mer based workflows were in line with LDT performance parameters per CLIA requirements (Kozyreva et al., 2017).

Thorough validation of alignment- and k-mer based methods revealed comparable results for accuracy, within- and between run precision, as well as analytical sensitivity/specificity. Only minor differences were observed between both approaches, with the k-mer based workflow achieving slightly higher values for repeatability, reproducibility and accuracy of AMR marker detection (in the range of 0.1–0.2%, non-significant). Detailed analyses for taxonomic identification revealed different bacterial species and LODs between the applied assays (16S rRNA identification, MLST, rMLST, and Kraken2). For the validation study we assembled representative strains of clinically relevant human pathogens, including among others a *Klebsiella pneumoniae* strain, which is a selected AST control for extended-spectrum beta-lactamase (ESBL) production (Clinical and Laboratory Standards Institute [CLSI], 2016). However, rMLST and Kraken2 analysis of the validation sequence and the reference sequence assigned the strain to *Klebsiella quasipneumoniae*. Subsequent investigation revealed a reclassification of ATCC^®^ 700603 to *Klebsiella quasipneumoniae* subsp. *similipneumoniae* (Maatallah et al., 2014; Elliott et al., 2016). A similar finding was that the tested *Enterobacter cloacae* isolate was confirmed by MLST and 16S rRNA identification, but assigned it to *Enterobacter hormaechei* by rMLST and Kraken 2. We assume that prior taxonomic assignment of the reference genomes was based on 16S rRNA annotations. Subsequently, extracting information from whole genome data via allele based rMLST (53 loci) and k-mer based Kraken2 indicates superior resolution in contrast to classical MLST (6–8 loci) and 16S rRNA (single loci) identification. This finding confirms the accuracy and high resolution of k-mer based typing and demonstrates the importance of constant database curation.

While it has been shown that NGS-based typing and AMR gene detection can be validated in line with CLIA guidelines (Kozyreva et al., 2017), we here demonstrate for the first time that k-mer based MLST and AMR marker detection reach performance characteristics in line with CLIA requirements. For MLST, sensitivity and specificity were 100% independent of the bioinformatics analysis approach. AMR marker detection, sensitivity and specificity were 95.29 and 99.78% for the k-mer based workflow compared to 95.17 and 99.77% for the alignment-based approach. Based on the high sensitivity and specificity for k-mer based AMR marker detection demonstrated in this study, we anticipate that analytical validation of AST prediction using k-mer based workflows and curated reference databases such as ARESdb as well as further k-mer based clinical microbiology assays can be shown in additional validation studies. The potential of direct prediction from genotype to phenotype has been shown repeatedly (Gordon et al., 2014; Bradley et al., 2015; Clausen et al., 2016; Pesesky et al., 2016; Tamma et al., 2019; Volz et al., 2019; Ferreira et al., 2020) and will likely continue to improve by the expansion of WGS databases combined with a better understanding of AMR mechanisms. Further, forcritical infectious diseases such as bloodstream infections, rapid identification of the causative pathogen as well as its resistance pattern is crucial for early optimization of the antimicrobial treatment regime (Grumaz et al., 2016). Previous studies already describe the potential of NGS as a culture-free method of analyzing the entire microbial community within a sample, including difficult to culture pathogens. Direct sequencing from clinical samples could reduce time and improve diagnostic value and patient outcome. Challenges, however, include diagnostic sensitivity, optimized workflows and data analysis (Doughty et al., 2014; Hasman et al., 2014; Long et al., 2016; Horiba et al., 2018; Brown and Christiansen, 2019). The described workflow is currently offered as end-to-end isolate characterization service for research use only via our own NGS service laboratory (Vienna, Austria). In addition to the validation study described here, we are currently working on making dry-lab workflows accessible on *ares-genetics.cloud* (for research use only), which should further enable laboratories to establish capabilities for NGS-based isolate characterization while not having to establish bioinformatics capabilities.

Based on our findings, we conclude that the developed k-mer based workflow enables reliable taxonomic classification, subtyping, phylogeny and AMR marker detection at high sensitivity and specificity. Thorough benchmarking of the k-mer based workflow revealed analytical performance criteria are comparable to alignment based workflows across all clinical microbiology assays evaluated.

## Data Availability Statement

The validation dataset has been deposited at the NCBI under bioproject accession PRJNA628576. Raw sequence data for each validation sample were deposited at the Sequence Read Archive (SRA) under accession numbers: SRR11614264–SRR11614298.

## Author Contributions

SL, SB, JW, and AP contributed to the conception and design of the study. SL, KM, and JW performed the experiments. SL, TW, SB, and AP analyzed the data. SL, TW, SB, JW, and AP wrote the manuscript. All authors contributed to the article and approved the submitted version.

## Conflict of Interest

SL, TW, KM, SB, and JW were employees of Ares Genetics GmbH. AP was Chief Executive Officer of Ares Genetics GmbH.

## Footnotes

1 http://broadinstitute.github.io/picard

2 https://github.com/samtools/bcftools

3 https://pubmlst.org/databases/

4 https://bigsdb.web.pasteur.fr/

5 https://github.com/tseemann/mlst

6 https://pubmlst.org/rmlst/

7 http://www.genomicepidemiology.org

8 https://github.com/tseemann/snippy.git

9 https://github.com/lh3/seqtk
