# Supplementary figures and images for: Analytical Performance Validation of Next-Generation Sequencing Based Clinical Microbiology Assays Using a K-mer Analysis Workflow
Source: Front Microbiol. 2020 Aug 5;11:1883. doi: 10.3389/fmicb.2020.01883 (PMC7422695; doi:10.3389/fmicb.2020.01883)

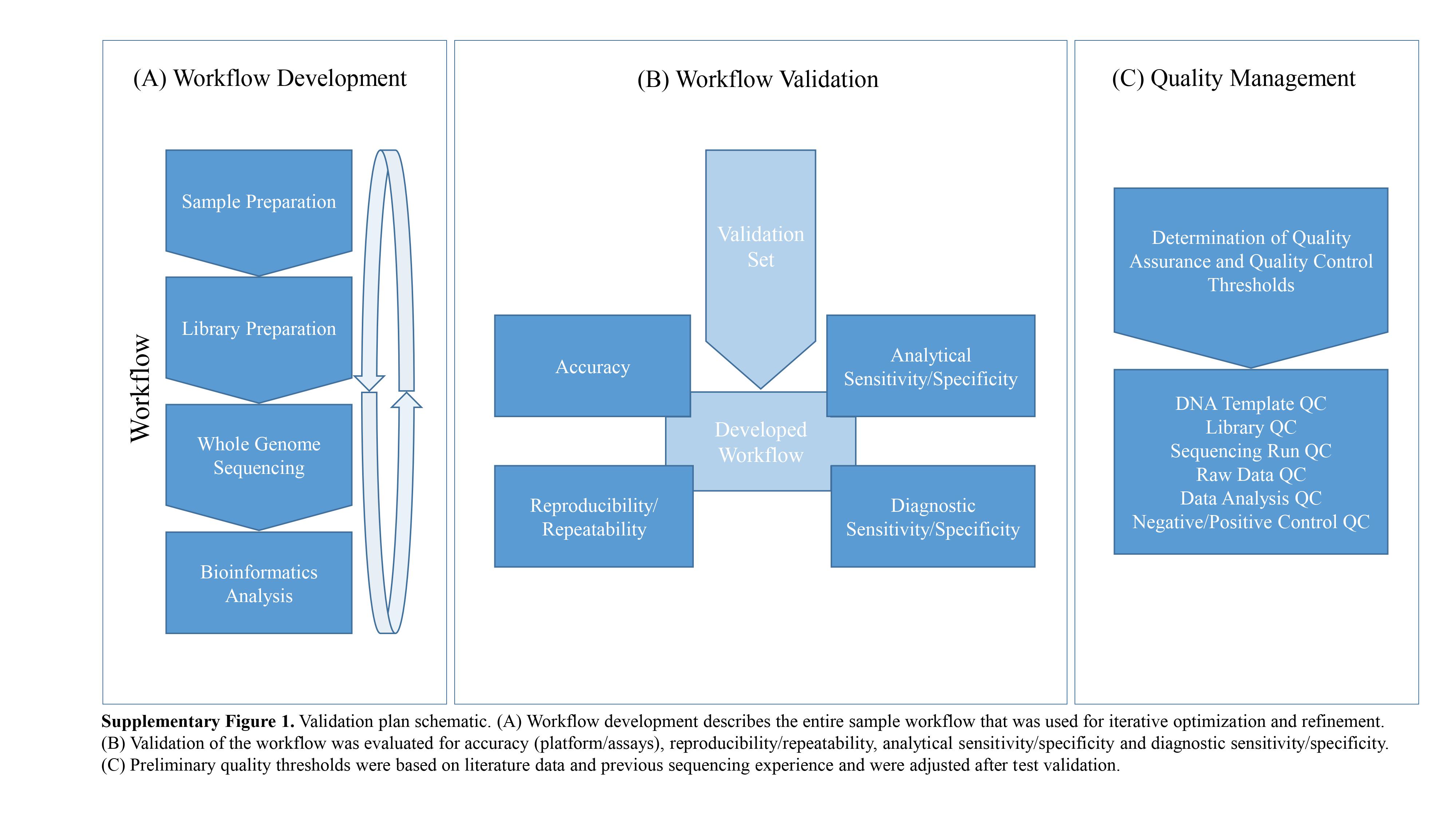

Supplement: Supplementary file 1 [file Image_1.JPEG]

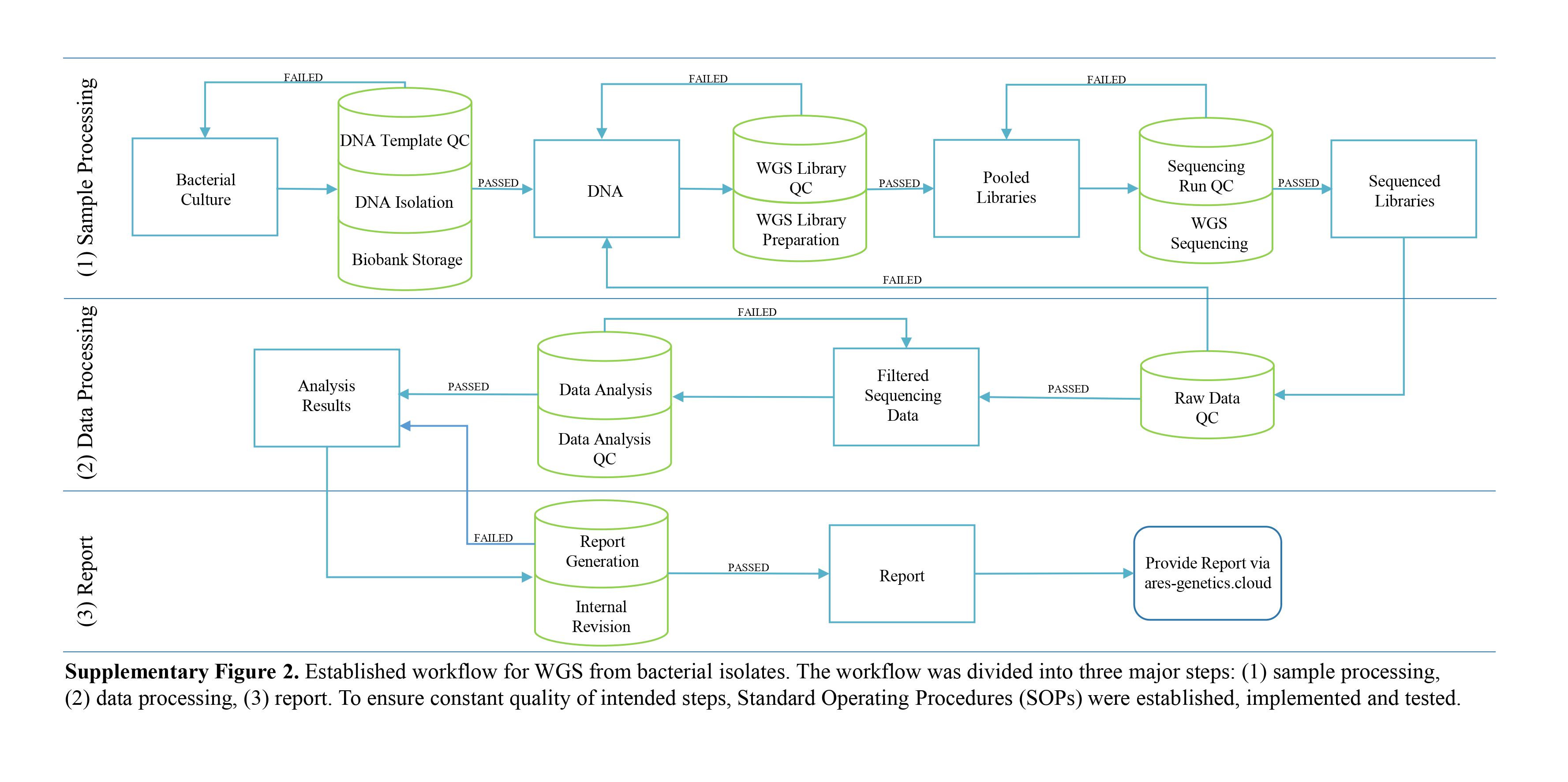

Supplement: Supplementary file 2 [file Image_2.JPEG]

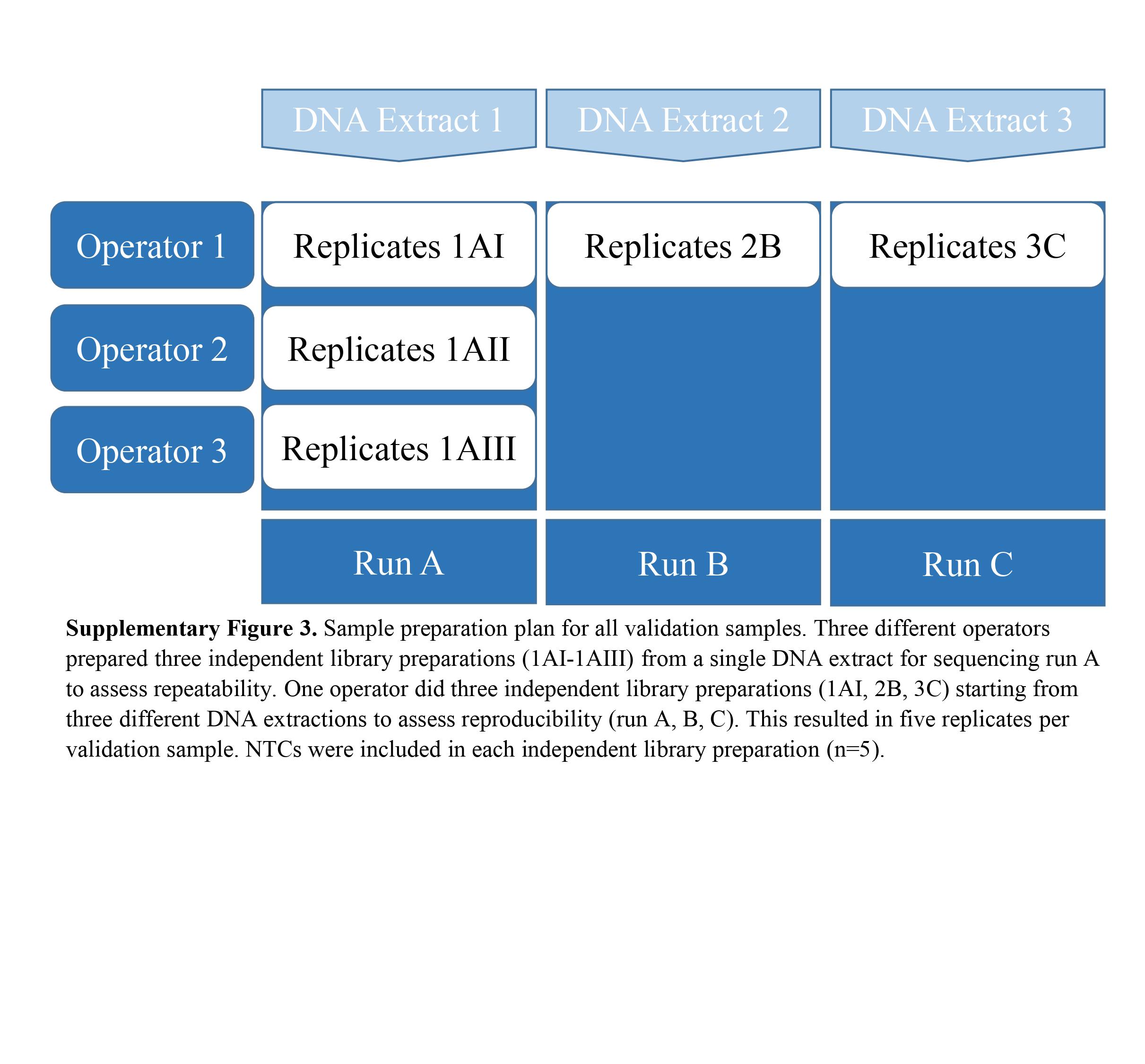

Supplement: Supplementary file 3 [file Image_3.JPEG]

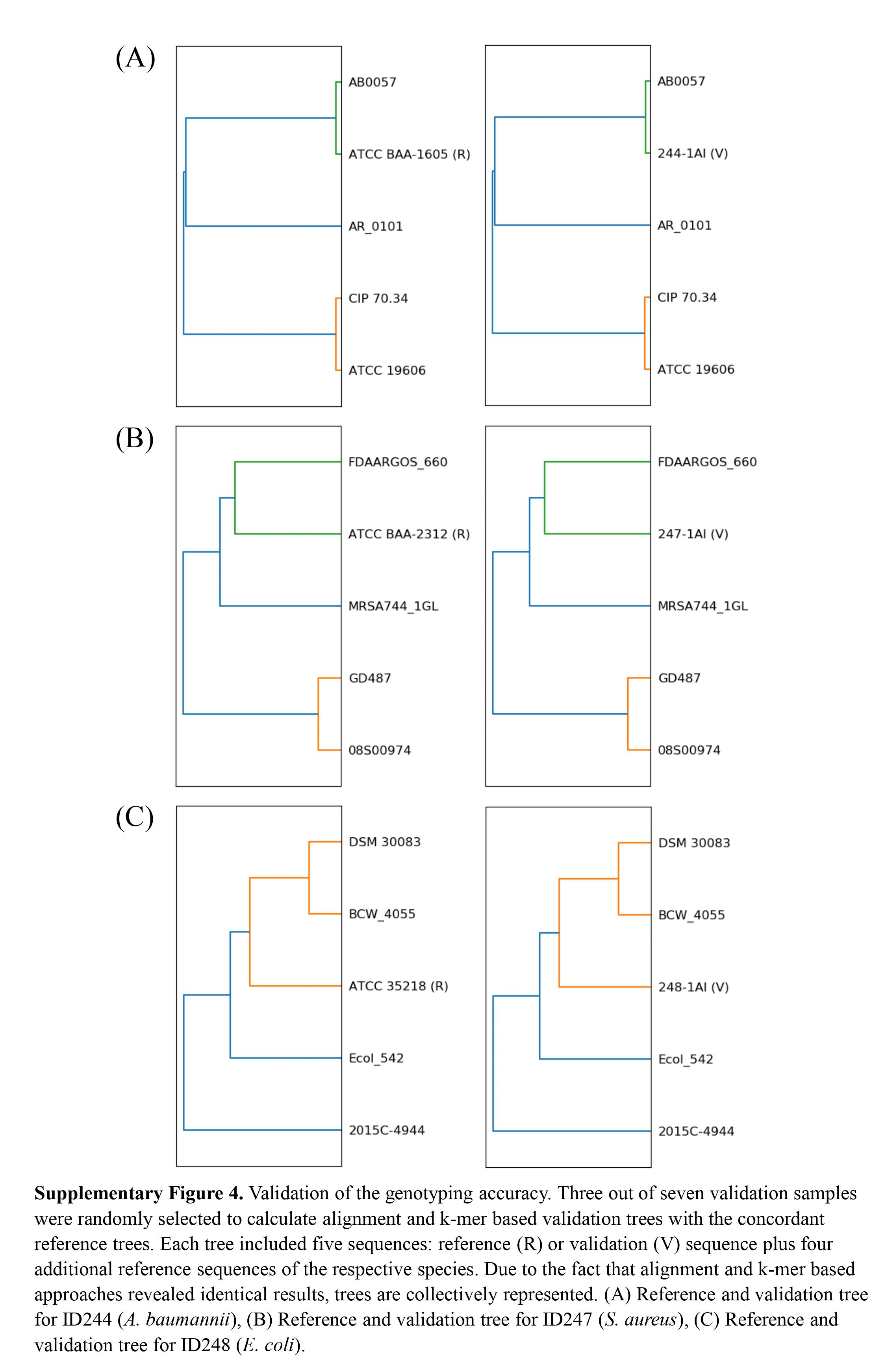

Supplement: Supplementary file 4 [file Image_4.JPEG]
